# Automatic Detection of Post-Operative Clips in Mammography Using a U-Net Convolutional Neural Network

**DOI:** 10.3390/jimaging10060147

**Published:** 2024-06-19

**Authors:** Tician Schnitzler, Carlotta Ruppert, Patryk Hejduk, Karol Borkowski, Jonas Kajüter, Cristina Rossi, Alexander Ciritsis, Anna Landsmann, Hasan Zaytoun, Andreas Boss, Sebastian Schindera, Felice Burn

**Affiliations:** 1 Institute of Radiology, Cantonal Hospital Aarau, 5001 Aarau, Switzerland; hasan.zaytoun@ksa.ch (H.Z.); felice.burn@ksa.ch (F.B.); 2 Institute of Diagnostic and Interventional Radiology, University Hospital Zurich, 8091 Zurich, Switzerlandkarol.borkowski@b-rayz.ch (K.B.);; 3 Institute of Diagnostic and Interventional Radiology, University Hospital Basel, 4031 Basel, Switzerland

**Keywords:** diagnostic imaging, post-operative clips, calcifications, breast neoplasms, artificial intelligence, deep learning

## Abstract

Background: After breast conserving surgery (BCS), surgical clips indicate the tumor bed and, thereby, the most probable area for tumor relapse. The aim of this study was to investigate whether a U-Net-based deep convolutional neural network (dCNN) may be used to detect surgical clips in follow-up mammograms after BCS. Methods: 884 mammograms and 517 tomosynthetic images depicting surgical clips and calcifications were manually segmented and classified. A U-Net-based segmentation network was trained with 922 images and validated with 394 images. An external test dataset consisting of 39 images was annotated by two radiologists with up to 7 years of experience in breast imaging. The network’s performance was compared to that of human readers using accuracy and interrater agreement (Cohen’s Kappa). Results: The overall classification accuracy on the validation set after 45 epochs ranged between 88.2% and 92.6%, indicating that the model’s performance is comparable to the decisions of a human reader. In 17.4% of cases, calcifications have been misclassified as post-operative clips. The interrater reliability of the model compared to the radiologists showed substantial agreement (κ_reader1_ = 0.72, κ_reader2_ = 0.78) while the readers compared to each other revealed a Cohen’s Kappa of 0.84, thus showing near-perfect agreement. Conclusions: With this study, we show that surgery clips can adequately be identified by an AI technique. A potential application of the proposed technique is patient triage as well as the automatic exclusion of post-operative cases from PGMI (Perfect, Good, Moderate, Inadequate) evaluation, thus improving the quality management workflow.

## 1. Introduction

Female breast cancer is the most diagnosed cancer in the normal population, with an incidence of 11.6%, as well as the second most common cause of death by cancer worldwide. More than two million cases of breast cancer and over 626,679 related deaths were registered in 2018 [1]. In the last decades, more and more countries have implemented an organized mammography screening program to detect malignant abnormalities of the female breast with great effort. For example, the International Agency for Research on Cancer (IARC) deduced data on breast cancer screenings and concluded that a 35% reduction in mortality can be observed in countries providing a common mammography screening program to women in the age range of 50–69 years [2].

As a result of technical development, mammography imaging shows a median sensitivity of up to 95% and a specificity of 96% [3], while the interpretation accuracy strongly depends on the image quality [4,5]. To ensure high-quality mammography images, the United Kingdom Mammography Trainers Group, with support from the Royal College of Radiographers, developed the PGMI (Perfect, Good, Moderate, Inadequate) assessment method [6]. This four-scale approach evaluates nine criteria for both cranio-caudal (CC) and medio-lateral oblique (MLO) views, including full breast tissue imaging, accurate exposure, proper compression, absence of motion, correct processing, absence of artifacts, absence of skin folds, and image symmetry. Images meeting all criteria are perfect; those deviating are rated as good, moderate, or inadequate. Operated breasts or those with artifacts are typically excluded from evaluation. Images of operated breasts or those with artifacts are typically excluded from PGMI evaluation when radiological technicians submit their personal reports for certification.

Accurate interpretation of mammographic or tomosynthetic images necessitates significant experience, expertise, and a systematic approach to ensure precise diagnosis and effective communication with patients, particularly during the post-operative follow-up phase after breast cancer treatment [7,8]. Post-operative clips play a crucial role in identifying surgical sites and highlighting the potential necessity for additional imaging modalities, such as ultrasound, to rule out residual or recurrent cancerous lesions in the previous tumor bed. However, in daily practice, these clips may be easily overlooked or misinterpreted, especially when captured in an orthogonal position and mistaken for calcifications. Hence, there is a pressing need for a technique enabling observer-independent detection of clip material to promptly signal the requirement for further diagnostic assessment of post-operative breasts, thereby optimizing patient risk stratification.

Utilizing machine learning techniques, such as the application of a U-Net, can serve this need and offer a pathway for implementing standardized classification methods such as triaging high-risk patients and optimizing workflow in quality control, such as automatically excluding cases for PGMI evaluation [9]. While deep Convolutional Neural Networks (dCNNs) have proven effective in analyzing medical images, there has been ongoing evaluation of various deep learning algorithms over time. For instance, a dCNN has demonstrated successful classification of mammographic images based on breast density according to ACR BI-RADS criteria [10]. In a separate study, the same dCNN was utilized to accurately detect and classify breast lesions in ultrasound imaging, achieving high accuracy [11]. Despite advancements in automated detection methods, challenges in postoperative clip detection in mammography persist due to image variability and artifacts. Traditional machine learning methods often struggle with complex patterns in medical images, whereas deep learning, particularly convolutional neural networks (CNNs), shows promise by learning features directly from data. Among CNN architectures, U-Net stands out for its effectiveness in biomedical image segmentation, capturing both local and global contexts. The adoption of a U-Net architecture, in particular, has emerged as a promising approach for processing medical image data [12]. Therefore, we categorized 1401 mammographies and tomosynthetic images into three groups: ‘background’, ‘calcifications’, and ‘post-operative clips’, based on annotations by an experienced radiologist. 

The objective of this retrospective cohort study was to assess the performance of a U-Net model in detecting post-interventional clip materials, aiming to develop an automated, observer-independent clip-detection system and deliver one of the first commercially available clip-detection tools on the market. Therefore, our study goal is to establish a pathway for the automatic detection of post-operative breast abnormalities using an AI approach, ultimately enhancing patient risk assessment and quality control.

The paper is structured in a Materials and Methods section, where datasets, preprocessing steps, the U-Net model architecture, and statistical analysis used in this study are explained; a Results section comparing AI and human annotations; and a Discussion that summarizes the key contributions and the impact of our proposed method on clinical practice, discusses the implications of our findings, the limitations of our study, and potential areas for future research.

## 2. Materials and Methods

### 2.1. Training and Validation Dataset

**Figure 1:**
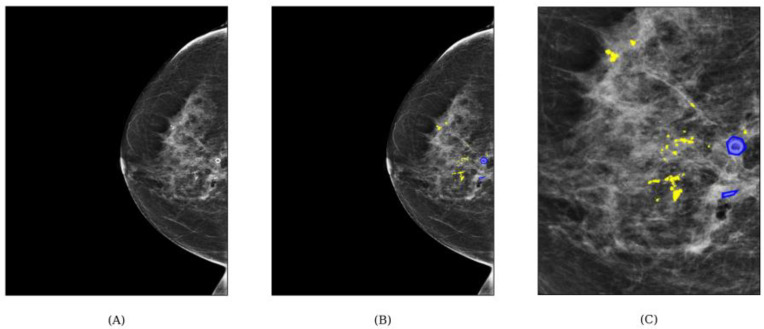
Exemplary visualization of ground truth (GT) labels. (**A**) shows the original image depicting two post-operational clips and many calcifications. (**B**) shows the segmentation labels after applying the post-processing steps (point-growing and high-contrast detection). A magnification of the precise labels is depicted in (**C**).

This retrospective study was approved by the local ethics committee (Ethics Committee Zurich, ID: 2016-00064). 819 2D full-field mammograms and 497 2D reconstructed tomosynthetic images were extracted. The inclusion criteria encompassed female patients with documented cases of calcifications or post-operative markings identified on mammography or tomosynthesis images. Due to the full anonymization of patient information, including patient ID and age, we cannot state the number of patients or median patient age. Two radiologists with 7 and 3 years of experience in breast imaging annotated the extracted data using an in-house-developed graphical user interface labeling tool. Precise segmentation labels were achieved by post-processing the annotations by the radiologists using point-growing and high-contrast-detection algorithms (Figure 1). Due to the post-processing steps, radiologists could time-efficiently annotate surgical clips and calcifications either as a point, from where a point-growing algorithm is applied, or as an area, wherein the high-contrast-detection algorithm searches for bright spots such as calcifications and clips. The DICOM images were converted to PNG format by using the values of the DICOM tags for window width and window height and then normalizing the pixel values to a range of 0 to 1. For training and validation, the dataset was split into two parts: 70% of the images were used to train the dCNN and 30% to evaluate the model.

### 2.2. Test Dataset

Additionally, a test dataset consisting of 85 images, making up 6% of the complete dataset, of 50 patients was extracted from varying institutions and annotated by two radiologists with 7 and 5 years of experience. The dataset included images not only from the institution represented in the training and validation sets but also encompassed a substantial portion of data sourced from three additional institutions, thereby representing three vendors of mammography and tomosynthesis devices (GE Healthcare, Chicago, ILUSA; Hologic, Marlborough, MA, USA and Siemens Healthineers, Erlangen, Germany). The inclusion criteria to the test dataset were identical to those applied for the training dataset. The dataset contains 65 2D full-field mammograms and 20 2D reconstructed tomosynthetic images. The annotation and post-processing pipeline was identical to that of training and validation, except for the annotators; one radiologist had not previously annotated training or validation data. The median patient age in the test dataset yielded 66.7 years. Of the test dataset, 61 images showed high resolution, 20 images showed low resolution, and 4 images showed unusually high contrast. The more experienced reader 1 served as a reference and labeled 79 images with respect to the presence of calcifications and surgical clips, while reader 2 labeled 45 images. Of these datasets, 39 images were separately annotated by both radiologists. 

### 2.3. Training of the dCNN

**Figure 2:**
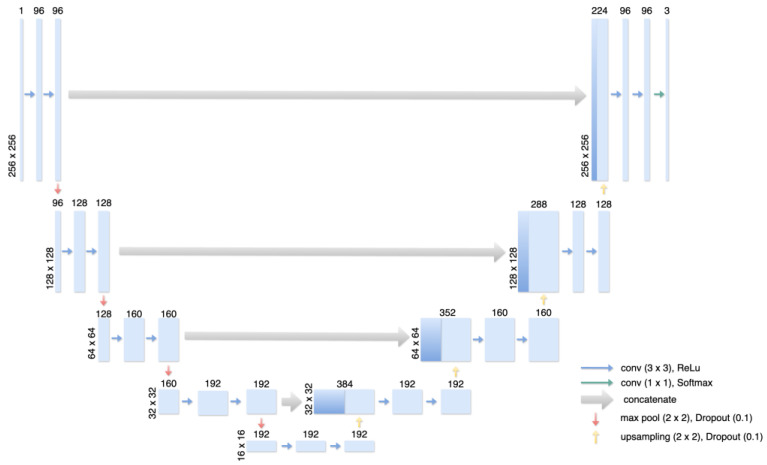
Illustration of the Multiclass U-Net-based Architecture. The encoder structure receives an input grayscale image of size 512 × 512 as input and consists of 4 encoder blocks with the filter sizes 96, 128, 160, and 192. Each block is built from two 2D convolutional layers with a kernel size of 3 × 3 and ReLU activation and is followed by batch normalization, max pooling for encoding, and dropout with a dropout rate of 0.1. The bottom of the U-shaped architecture consists of the same layers and properties as before but is followed by a 2D-up sampling layer and a dropout rate of 0.1. The decoder structure is built symmetrically to the encoder structure but receives two inputs: the output of its counter-encoder part and the output of the preceding decoder block. This way, information about different resolutions can be maintained. The last decoder block is followed by a final 2D convolutional layer with a kernel size of 1 × 1 and a filter size of 3 with Softmax activation, resulting in 3 output maps.

**Figure 3:**
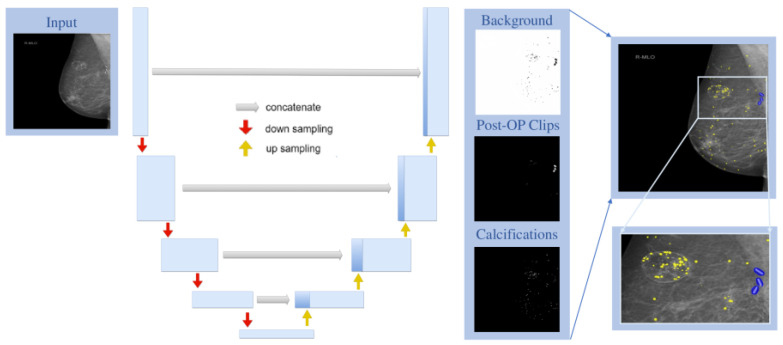
Schematic of the U-Net-based architecture: The U-Net receives a 512 × 512 resized image as input, which is processed through the encoder- and decoder structures of the network. The U-Net outputs three segmentation masks for background, post-operative clips, and calcifications. The detected clips and calcifications are then visualized by taking the maximum value of each class per pixel (yellow: calcifications, blue: post-operative clips).

A single segmentation dCNN based on the U-Net architecture [13] was implemented and trained for 45 epochs (Figure 2). The dimensions of the different convolutional layers, as well as the dropout, were adapted. As depicted in Figure 3, the network receives a 512 × 512 grayscale image as input and predicts three segmentation masks for background, surgical clips, and calcifications. The model was trained with a batch size of 2 using categorical cross-entropy loss and an Adam optimizer with an initial learning rate of 0.001, which is reduced by a factor of 10 each time the validation loss stalled for longer than five epochs. To ensure a diverse and robust dataset, data augmentation was performed in real-time during training. This included the application of zoom by a maximum of 10%, random rotation up to 10 degrees, horizontal and vertical flipping of the images, and a horizontal and vertical shift by a maximum of 10%. To reduce overfitting, a dropout of 0.1 was used both in the encoder and decoder structures of the dCNN. All computations were performed on an NVIDIA GeForce RTX 3090 GPU with 24,576 MiB (33 MHz) using Python 3.10.8 and Tensorflow 2.8.0.

### 2.4. Statistical Analysis

During training and validation, the model was evaluated using pixel classification accuracy and intersection over union (IoU).

(1) $$IoU=\frac{Area\;of\;Intersection}{Area\;of\;Union}$$

While these metrics are sufficient to measure model training and convergence, they only provide limited information about model performance. This is caused by the heavy class imbalance (background pixels make up more than 99% of the data) of the optimization problem in this study. Thus, the test data was evaluated by visual inspection, comparing the predictions of the model to the annotations of the two radiologists on single views, which served as ground truth (GT). Moreover, we compared both human readers to each other, where the annotations of the more experienced reader (reader 1) served as GT, and we compared the results of the model against human-labeled GT against the results between both human readers. True positives (TP), false positives (FP), and false negatives (FN) were counted for both post-operational clips and calcifications for all three cases (model vs. reader 1, model vs. reader 2, and reader 2 vs. reader 1). Note that calcifications were counted as a cluster if applicable. Moreover, correct classifications (true positives and true negatives) as well as misclassifications of surgical clips as calcification (false negatives (FN)) or conversely, calcifications as surgical clips (false positives (FP)) were counted to generate normalized confusion matrices and accuracy.

(2) $$Accuracy=\frac{TP+TN}{TP+TN+FP+FN}$$

Using Python 3.10.8 and the scikit-learn library (version 1.2.1), inter-rater reliability between the dCNN and two radiologists was assessed by computing Cohen’s Kappa κ, a robust statistic to evaluate the agreement of different readers. The Kappa results are interpreted as follows: values ≤ 0 as indicating no agreement, 0.01–0.20 as none to slight, 0.21–0.40 as fair, 0.41–0.60 as moderate, 0.61–0.80 as substantial, and 0.81–1.00 as almost perfect agreement (according to Landis & Koch, 1977) [14]. 

## 3. Results

### 3.1. Training and Validation 

The network converged after 45 epochs of training at a training and validation loss of 0.00181 and 0.00179, respectively. Moreover, both training and validation accuracy amounted to 99.95%, while training and validation IoU scores measured 99.84% and 99.85%, respectively. 

### 3.2. Clinical and Statistical Validation of the Test Dataset

The measured accuracies for detected clips, detected calcifications, and classification of detected clips and calcifications on the test dataset are shown in Table 1. The accuracy of the test dataset annotated by both readers (39 images) for detected clips measured 88.1% (model vs. reader 1) and 86.9% (model vs. reader 2). In comparison, reader 2 detected clips with an accuracy of 91.7% compared to reader 1. The highest values of TP (clips detected as clips) and TN (calcifications detected as calcifications) were achieved by the model compared to the more experienced reader (reader 1). The FN (missed clips) detections of the model were similar to human performance when compared to each other. FP (wrongly detected clips) occurrences in the model were significantly higher than human FP detections. For detected calcifications, the accuracy measured 83.7% (model vs. reader 1), 80.4% (model vs. reader 2), and 84.3% (reader 1 vs. reader 2).

**Figure 4:**
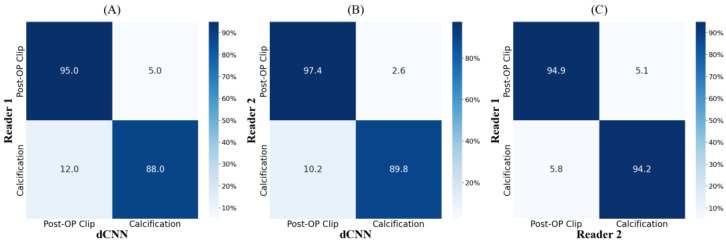
Confusion matrices showing the predictions of the defined classes post-operational clips and calcifications of the dCNN and two readers. (**A**,**B**) show the performance of the dCNN compared to readers 1 and 2, respectively. In comparison, the agreement of the two readers for classification is shown in (**C**).

**Figure 5:**
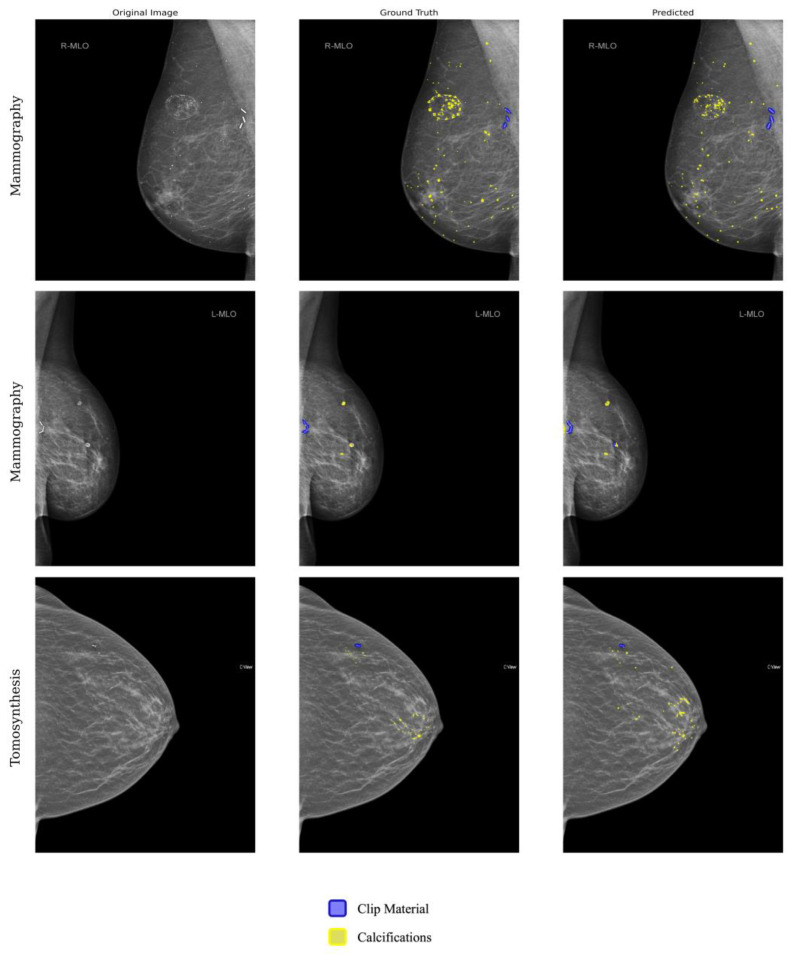
Top row: Exemplary model prediction of non-suspicious calcifications and post-operative clips in conventional 2D-full field mammography (left) original image with no annotation (middle) manually annotated image with marked calcifications (yellow) and marked clip material (blue) (right) automatically annotated image with marked calcifications (yellow) and marked clip material (blue). Middle row: Exemplary model prediction of non-suspicious calcifications and post-operative clips in conventional 2D-full field mammography (left) with no annotation (middle) manually annotated image with marked calcifications (yellow) and clip material (blue) (right) automatically annotated image with marked calcifications (yellow) and true-positive and false-positive marked clip material (blue). Bottom row: Exemplary model prediction of non-suspicious calcifications and post-operative clips in 2-D reconstructed tomosynthesis (left) original image with no annotation (middle) manually annotated image with marked calcifications (yellow) and marked clip material (blue) (right) automatically annotated image with marked calcifications (yellow) and marked clip material (blue).

The confusion matrices for the classification of detected clips and calcifications for the model and both readers are shown in Figure 4. 95% and 97.4% of the clips were classified correctly by the model with respect to readers 1 and 2, with 5% and 2.6% of the clips being misclassified as calcifications. The results achieved by the human readers are slightly lower, with 94.9% of the clips detected correctly and 5.1% of misclassified clips. The model misclassifies calcifications as clips with 12% (model vs. reader 1) and 10.2% (model vs. reader 2) more often than the human reader with 5.1% (reader 1 vs. reader2). The number of TP, TN, FP, and FN resulted in classification accuracies for detected clips and calcifications that are shown in Table 1 and amounted to 89.5% (model vs. reader 2), 91.4% (model vs. reader 2), and 94.3% (reader 1 vs. reader 2). Figure 5 shows exemplary results for the model prediction and distinction of calcifications and post-operative clips in tomosynthetic images as well as in mammography images. Both the radiologists and the algorithm revealed a low rate of false negative cases, resulting in 6 (model vs. reader 1), 4 (model vs. reader 2), and 6 (reader 1 vs. reader 2) missed clips. The lowest value of the kappa (κ = 0.74) was obtained for the reliability of reader 1 and the reference, while the highest one (κ = 0.78) was obtained for reader 2. Both readers, compared to each other, showed near-perfect agreement (κ = 0.84). 

## 4. Discussion

In the present study, we propose an approach for the fully automatic detection of post-interventional clip material in 2D mammography images as well as tomosynthesis using a U-Net dCNN and compare its performance directly to the diagnostic performance of two experienced radiologists. 

The dCNN underwent training using a dataset consisting of 819 mammographies and 497 tomosynthesis scans. It achieved an overall classification accuracy ranging from 89.5% to 91.4% on the test dataset, evaluated against the annotations provided by readers 1 and 2, respectively. Comparatively, dCNN’s performance in detecting clip material closely resembles human-readout, with 87.0% to 88.1% accuracy in correctly classifying clips. However, the model exhibits a higher rate of false positives (17 and 20 false positives compared to readers 1 and 2, respectively), in contrast to the human readout, which yielded only 9 false positives between the two readers. The observed phenomenon can be attributed to the limited number of training images (*n* = 922) and the subsequent small variability in calcifications in the training dataset. As the test dataset contains images from varying institutions, an additional domain shift might contribute to the falsely positive predictions. Moreover, the ability of human readers to interpret the entire breast tissue comprehensively, including detecting various post-operative changes such as scarring, may be an additional factor. Consequently, while a human reader may identify the lack of postoperative changes, a deep convolutional neural network (dCNN) may be more inclined to classify calcifications as clips. 

In general, the diagnostic quality strictly depends on the image quality. Beside movement artifacts, the quality of 2D mammographies as well as tomosynthesis can be heavily influenced by metal implants, such as implantable cardioverter-defibrillator (ICD) or post-biopsy clips. In both 2D mammography and, especially, tomosynthesis, the appearance of clip material can lead to artifacts. A very common phenomenon is the so-called “slinky artifact”, which is characterized by a train of low attenuation ripples underlying dense foreign material, such as metal. In the test dataset, 24 images showed “uncanny contrast” or “low resolution” and can be derived from 2-D reconstructed tomosynthesis. Those kinds of artifacts appear due to the inability to suppress (out-of section) noise when the number of acquired projections is less than the intended reconstruction sections. The model missed the clip in only 4 (reader 1) and 3 cases (reader 2) while misclassifying 17 (reader 1) and 20 localizations (reader 2) with underlying dense regular tissue or calcification as clip material. The higher amount of false positive clips compared to the number of missed clips is possibly related to the image acutance and intensification of slinky artifacts of tomosynthesis in a dataset where conventional mammography images and 2D reconstructed images were mixed [15]. However, false positives in this context are typically benign, leading to non-invasive confirmatory imaging rather than unnecessary interventions. In post-operative settings, a higher false positive rate is acceptable, as it ensures that fewer true positives are missed. This is critical for the early detection of complications or residual material, thereby enhancing patient safety.

For both groups, the human readers as well as the dCNN algorithm, the differentiation of slinky artifacts and calcifications can be a challenging task, thus leading to misclassification. Another possible reason for the false positive results can derive from the fact that some round calcifications were interpreted as orthogonal captured clips. Due to the missing second projection in the test dataset, a single bright dot resulting from calcification can mimic a perpendicular captured clip. Nevertheless, the performance of our algorithm is adequate with respect to the low number of training images.

The rate of false negative cases (reader 1 vs. model = 6 false negatives, reader 2 vs. model = 4 false negatives) is comparable to the human readout (reader 1 vs. reader 2 = 6 false negatives). Both readers are experienced radiologists in the field of breast imaging, which is why the classification accuracy especially for the differentiation between clips and calcifications, only varied slightly. These small deviations in classification and reference are also reflected in the interrater reliability assessed in our study, which showed a “near perfect” correlation. 

There are several reasons for marking a breast with clip material. Clips can be implemented when taking a biopsy of a suspect breast lesion, or they appear in post-operative breasts, marking the former tumor bed [16,17,18]. In both cases, our algorithm will detect the clip and indicate the potential need for additional ultrasound, as the former tumor bed represents the area most likely to be affected by a relapse [19]. In case the algorithm detects a post-biopsy clip, the attending radiologist may decide against an additional examination after checking the clinical history of the patient carefully. The AI identification of post-operative foreign material with the developed algorithm has the potential to enable optimization of patient triage and could lead to a faster evaluation of post-operative breasts with additional imaging techniques such as ultrasound. This can reduce the recall rates of patients either in a screening or a curative setting, resulting in less psychological stress for the patient and fewer costs for the mammography facility.

Furthermore, this algorithm can be used as an independent classification tool and automatically exclude post-operative cases from PGMI evaluation, thus accelerating the workflow for the radiological technician. Operated breasts and breasts showing artifacts are normally excluded from the PGMI evaluation when the radiological technician hands out their personal report of taken images for certification. The developed algorithm helps to automate this process while excluding patients with previous breast surgery or breasts showing artifacts. Likewise, the algorithm is applicable to other quality control systems, such as, for example, EAR, a three-scale control system (excellent, adequate, repeat) [20].

In the following, some limitations of this study are discussed. First, the anonymization of the patient cohort and consequently the lack of patient information must be considered limitations, that restrict the understanding of the dataset’s diversity. Secondly, the performance of the dCNN was compared with the performance of two radiologists, who read the images in a completely blinded manner. In order to provide an objective comparison between the human readers and the dCNN, it was necessary for the radiologists to classify the images in a machine-like manner without seeing any second projections or knowing any further information. This, of course, does not reflect the real workflow in the clinical routine, as real-life radiologists are aware of the complete image data of the patient as well as of previous examinations, interventions, and complementary imaging data. Moreover, during the evaluation of our model on the test dataset, the annotations of the respective experienced radiologist served as ground truth instead of radiological reports and findings. This way, local information about calcifications and clips could be used; however, the annotations made by the radiologists can be flawed.

An additional constraint arises from the utilization of clips to delineate the tumor in patients undergoing neoadjuvant chemotherapy. In scenarios where patients remain eligible for radiotherapy post-neoadjuvant therapy, clip placement at the tumor site remains relevant. However, for patients undergoing intraoperative radiotherapy, bypassing external radiotherapy, the necessity for clip marking diminishes, or the clip itself may be removed during Breast Conserving Surgery (BCS) following neoadjuvant chemotherapy [21,22]. This variance in practice needs to be addressed and emphasizes that the non-presence of clips does not rule out previous cancer. While the algorithm will not be able to identify the post-operative status in these patients, the attending radiologists still have to study the patient’s clinical history, if available, to ensure an accurate diagnostic procedure. 

However, the main limitation of this study was the low number of images. We were able to reach high accuracy with our dCNN algorithm. Furthermore, our test dataset consisted of both internal and external data acquired from devices from three different manufacturers. Given the high variance in our test dataset, it was shown that the augmentation used in training was sufficient to make the model robust to varying vendors. Nonetheless, further annotation will be needed to increase the number of correctly classified clips. The variable appearance of different clip brands as well as variations in the clip length of the same manufacturer can decrease the accuracy of our algorithm and have to be investigated in further studies with a higher number of images. Moreover, one significant challenge is the issue of pixel imbalance, where background pixels vastly outnumber the pixels representing the regions of interest. To address this, implementing focal loss could be beneficial, as it focuses on hard-to-classify examples and mitigates the effect of class imbalance. Additionally, exploring alternative architectures that do not rely on segmentation, such as object detection networks, might offer enhanced performance and efficiency. With this study, we show that post-interventional clips can be adequately identified by an AI. Moreover, its use for patient triage in the AI era, the developed algorithm can be used for quality control and can serve as an objective tool for the automated exclusion of post-operative cases from PGMI evaluation and improving the workflow of the technician.

## Figures and Tables

**Table 1:** Accuracy, true positives (TP), true negatives (TN), false positives (FP), and false negatives (FN) of detected post-operational clips, calcifications, and both clips and calcifications for the different readers and dCNN. The accuracy of detected clips and calcifications was calculated by counting TP, FP, and FN for the given class. True negative (TN) occurrences of the opposite class being correctly detected were counted. The best-achieved scores, both the lowest and highest, are marked in bold.

	Accuracy of Detected Clips				Accuracy of Detected Calcifications				Classification Accuracy of Detected Clips and Calcifications			
dCNN/Reader 1	88.1%				83.74%				89.5%			
dCNN/Reader 2	87%				80.4%				91.4%			
Reader 1/Reader 2	**91.7%**				**84.3%**				**94.3%**			
	**TP**	**TN**	**FP**	**FN**	**TP**	**TN**	**FP**	**FN**	**TP** **(Clips)**	**TN** **(Calcifications)**	**FP** **(Calcification detected as Clip)**	**FN** **(Clip detected as Calcification)**
dCNN/Reader 1	**38**	**132**	17	6	132	**38**	5	28	**38**	**132**	18	2
dCNN/Reader 2	37	123	20	**4**	123	37	22	**17**	37	123	14	**1**
Reader 1/Reader 2	37	129	**9**	6	**129**	37	**3**	28	37	129	**8**	2

## Data Availability

The raw data supporting the conclusions of this article will be made available by the authors on request.
